# *Moraxella tarda* sp. nov., a Novel Species Isolated from Nasal Cavity of Cattle with Infectious Bovine Keratoconjunctivitis

**DOI:** 10.1007/s00284-026-05050-6

**Published:** 2026-07-09

**Authors:** Robert Domingues, Clarissa Vidal de Carvalho, Daniele Ribeiro de Lima Reis Faza, Helena Brocardo Comin, Newton Valério Verbisck, Alessandra Figueiredo de Castro Nassar, Wanessa Araújo Carvalho, Fernando Flores Cardoso, Alessandra Barbosa Ferreira Machado, Marta Fonseca Martins, Emanuelle Baldo Gaspar

**Affiliations:** 1 Embrapa Southern Livestock, Bagé, RS Brazil; 2 Universidade Federal de Juiz de Fora, Juiz de Fora, MG Brazil; 3 Embrapa Dairy Cattle, Juiz de Fora, MG Brazil; 4 Universidade Federal do Pampa, Dom Pedrito, RS Brazil; 5 Embrapa Beef Cattle, Campo Grande, MS Brazil; 6 Biological Institute, São Paulo Agency for Agribusiness Technology, Secretary of Agriculture and Food Supply, São Paulo, SP Brazil

## Abstract

A novel bacterial species was isolated from the nasal mucosa of Hereford cattle exhibiting early clinical signs of infectious bovine keratoconjunctivitis on a beef cattle farm in the Pampa biome, southern Brazil, in 2018. Cells were Gram-stain-negative cocci and exhibited slow growth under standard laboratory conditions, with optimal growth at 35 °C, pH 7.5, and 1.0% NaCl. The draft genome comprised approximately 1.86 Mbp (GenBank accession number GCF_035181365.1). Phylogenetic analysis based on the 16 S rRNA gene sequence showed that the closest relative was ar*Moraxella nasibovis*, with 97.9% sequence identity. Genome-based comparisons revealed average nucleotide identity (ANI) values ranging from 77% to 79% and digital DNA–DNA hybridization (dDDH) values ranging from 20.5% to 33.7% when compared to other *Moraxella* species, all below the accepted thresholds for species delineation. Phylogenomic analyses based on whole-genome and core gene datasets consistently placed the isolate in a distinct lineage within the genus *Moraxella*, most closely related to *M. nasibovis* and *M. oculi*. Matrix-assisted laser desorption/ionization time-of-flight mass spectrometry (MALDI-TOF MS) did not match the isolate with any known *Moraxella* species. Morphological, physiological, and genomic analyses supported its classification within the genus *Moraxella* while clearly distinguishing it from previously described species. Based on the combined phenotypic and genomic evidence, we propose the novel species as *Moraxella tarda* sp. nov., with strain 7624LNᵀ (= CCP 570ᵀ = IAL 11705ᵀ = LMG 34122ᵀ) designated as the type strain.

**Supplementary Information:**

The online version contains supplementary material available at https://doi.org/10.1007/s00284-026-05050-6.

## Introduction

*Moraxella* is a genus of Gram-negative, catalase- and oxidase-positive bacteria. Members of the genus exhibit pleomorphism, ranging from cocci to rod-shaped forms. Currently, the genus *Moraxella* comprises 25 species with validly published name, according to the List of Prokaryotic Names with Standing in Nomenclature (LPSN; accessed on 08 May 2026) [1], isolated from mammals [2]. Common isolation sites for *Moraxella* species include the ocular conjunctiva, oral cavity, and upper respiratory tract.

Despite its clinical and ecological relevance, the taxonomy of the genus remains complex and historically unstable. Recent advances in genome-based taxonomy have demonstrated that the genus *Moraxella* has historically exhibited polyphyly and remains taxonomically complex, with distinct phylogenetic lineages requiring revision. In this context, comprehensive taxogenomic analyses integrating core-genome phylogeny, average amino acid identity (AAI), and percentage of conserved proteins (POCP) have led to the reclassification of several species previously assigned to *Moraxella*. Notably, *Moraxella boevrei*, *Moraxella osloensis*, and *Moraxella atlantae* have been reassigned to the genus *Faucicola*, while *Moraxella lincolnii* has been proposed as a member of a novel genus, *Lwoffella* [3]. These findings highlight the impact of genomic approaches in refining bacterial systematics. The use of genome-based analyses has provided a reliable and highly informative framework for inferring phylogenetic relationships and has supported the classification, identification, and delineation of prokaryotic taxa [4].

Among bovine-associated species, *Moraxella bovis* stands out as the most extensively investigated species. It is primarily linked to the manifestation of infectious bovine keratoconjunctivitis (IBK), a prevalent ocular ailment affecting cattle worldwide. Nevertheless, various other species within the genus *Moraxella* have been identified in both IBK-affected and non-affected bovine populations. Noteworthy among these are *Moraxella bovoculi* [5] and, more recently, *Moraxella oculi* [6] and *Moraxella nasibovis* [7]. The precise role of these additional *Moraxella* species in the pathogenesis of IBK and other bovine diseases remains uncertain [8–10].

In a previous study, 55 isolates of *Moraxella bovis* and *Moraxella bovoculi* were collected from nasal and ocular swabs obtained from Hereford steers in four farms situated in the southern region of the State of Rio Grande do Sul, Brazil, within the Pampa biome. However, during the analysis of those isolates, specifically focusing on the DNA sequence of the 16–23 S rRNA intergenic region, certain isolates did not match as any known *Moraxella* species. Those isolates remained categorized as *Moraxella* sp [11].

The aim of this study was to characterize the genomic features, MALDI-TOF protein mass profile, and physiological traits of this novel *Moraxella* species. The comparative molecular analyses conducted showed that these microorganisms are clearly distinguished from other species within the genus *Moraxella* and are representative of a new species. Specifically, the strain 7624LN^T^ has been selected as the type strain, and therefore, we propose the name *Moraxella tarda* sp. nov. for this newly identified species.

## Methods

### Strain Isolation and Culture Conditions

Samples were collected from Hereford steers exhibiting initial clinical manifestations of infectious bovine keratoconjunctivitis (IBK) by gently swabbing the inferior conjunctival sac and nasal cavity. The samples were immediately seeded onto tryptone soy agar supplemented with 5% blood (TSAB) (Kasvi) and incubated at 37 °C for 48 h. Individual colonies displaying morphological characteristics compatible with species of the genus *Moraxella* (whitish, smooth, and small) were subsequently streaked onto fresh TSAB plates to obtain pure cultures.

Among the 55 *Moraxella* isolates obtained during that survey, three isolates exhibited morphological and biochemical characteristics consistent with the genus *Moraxella* but could not be assigned to any known species using PCR–RFLP analysis of the 16–23 S rRNA intergenic spacer (ITS) region, as they did not produce amplicons of sizes compatible with described taxa. Sequencing of the ITS region (i.e., the 16–23 S rRNA intergenic spacer, not the 16 S rRNA gene) revealed less than 92% similarity to any sequence available in public databases, although the closest matches corresponded to members of the genus *Moraxella*.

The novel taxon was recovered at a low frequency, representing approximately 1.58% (3 out of 55) of the *Moraxella* isolates obtained in this study. However, because bacterial isolation was performed by streaking ocular and nasal samples onto blood agar plates, where multiple bacterial species may grow and colony overlap may occur, the precise abundance of the novel taxon in the original samples cannot be accurately determined.

The isolates (later recognized as strains of the novel species) were named according to the identification number of the animal from which they were recovered, followed by the collection site (LN for left nostril and RN for right nostril). All data for strain 7624LN^T^ are publicly available in the NCBI database under BioSample accession SAMN39049954, genome assembly GCF_035181365.1, and 16 S rRNA gene sequence PP069797.1.

### Genome Sequencing, *de novo* Assembly and Annotation

The strain 7624LN^T^ was subjected to DNA extraction by the method described in [12] and the genomic DNA was randomly fragmented by sonication, end polished, A-tailed, and ligated with the full-length adapters of Illumina sequencing, followed by further PCR amplification with P5 and indexed P7 oligos. The PCR products as the final construction of the libraries were purified with AMPure XP system (Beckman Coulter). Then libraries were checked for size distribution by Agilent 2100 Bioanalyzer (Agilent Technologies) and quantified by real-time PCR. The qualified libraries were sequenced using paired-end (2 × 150 bp) libraries in the Illumina Novaseq 6000 sequencer (Illumina), producing approximately 9.5 million reads for the isolate.

The sequencing data were uploaded to the Galaxy web platform, where quality control, trimming, and genome assembly were performed [13]. First, the quality of the reads was evaluated with the FastQC v. 0.74 [14] and trimmed with Trimmomatic v. 0.38.1 [15] using the parameters: *LEADING* = 8; *TRAILING* = 8; *SLIDINGWINDOW* = 4:22 and *MINLEN* = 50. After trimming, only the paired reads were retained for re-evaluation with FastQC and submitted to *de novo* assembly with SPAdes v. 3.15.4 [16] using the –careful option to reduce the number of mismatches and short indels, and K-mer size values of 21, 33, 55, 77 and 99. Assembled scaffolds were filtered manually according to their coverage (> 10x) and length (> 500 bp) to eliminate potential contaminants. Genome annotation was performed using the BV-BRC v. 3.32.13a platform [17], which provides standardized structural and functional annotation via the RASTtk tool [18] for bacterial genomes. Genome completeness was assessed using BUSCO v5.8.2 [19] in genome mode with the bacteria_odb10 dataset. Genome completeness and contamination were additionally evaluated using CheckM v1.2.4 [20] with the taxonomy workflow at the class level (Gammaproteobacteria).

### Comparative Genomics Analyses

The draft genome was compared to the reference genomes of all known *Moraxella* species available on GenBank/RefSeq (Table S1). Genomes from two recently defined genotypes of *M. bovis* [21] and *M. bovoculi* [22] were included. *Pseudomonas aeruginosa* was used as outgroup. These genomes were compared by the following methods: (I) average nucleotide identities (ANIs) estimated between each pair of genomes using the FastANI v.1.34 [23], implemented through the enveomics collection [24] with relaxed fragment fraction (minFraction = 0.1); (II) 16 S rRNA sequence-based and whole genome-based taxonomic analysis in the Type (Strain) Genome Server (TYGS) platform [25]; (III) in-silico genome-to-genome distance calculation (GGDC), method that mimics DNA-DNA hybridization (DDH), with the GGDC 3.0 tool [26]; (IV) Bacterial Genome Tree Service available on BV-BRC platform, which selects single-copy BV-BRC PGFams and analyzes aligned proteins and coding DNA from single-copy genes using the program RAxML [27], using the parameters: “max allowed deletions” = 3 and “max allowed duplications” = 3, which allowed the use of 789 single-copy genes. (V) phylogenomic analysis based on a concatenated core gene dataset using EasyCGTree v4.2 [28], which identifies conserved single-copy genes through profile HMMs. A maximum-likelihood tree was inferred using IQ-TREE under the supermatrix approach, and branch support was assessed using ultrafast bootstrap with 1,000 replicates.

### Morpho-physiological Characterization

Gram staining was performed by using a Gram stain kit (NewProv) following the manufacturer’s recommendations. Cell morphology was observed under optical microscopy (Olympus BX53) at 1000X magnification. Colony morphology, hemolytic activity and microaerophilic growth in candle jar were evaluated after seeding on TSAB and incubation at 35 °C for seven days. Growth on MacConkey agar (HiMedia) was performed at 35 °C for three days. For determination of optimal growth conditions, BHI broth (Kasvi) was used in combination with variations in temperature (4, 25, 31, 33, 35, 37, 39, 41, and 43 °C), pH (5.0–10.0 at 0.5-unit intervals), and NaCl concentration (0–3.0% w/v at 0.5% intervals). Optical density at 600 nm (OD600) was used as an indirect measure of bacterial growth.

Catalase activity was assessed by measuring the production of oxygen bubbles in a 3% hydrogen peroxide solution. Oxidase activity was determined by inoculating bacteria onto oxidase paper strips (LaborClin) in accordance with the manufacturer’s guidelines. To assess hydrogen sulfide production, indole formation and motility, bacteria were cultured in SIM medium (HiMedia). The nitrate reduction test involved the use of 0.1% (w/v) potassium nitrate in tryptone soy broth (TSB). Fermentation of glucose, lactose, sucrose, and mannitol was examined by introducing these carbohydrates at a concentration of 1.0% (w/v) into peptone water (HiMedia). Bacteria were cultured on solid medium containing skim milk (28%), tryptone (5%), yeast extract (2.5%), dextrose (1%), and agar (15%), for the casein hydrolysis test, and halo formation was evaluated after incubation. Bacteria were inoculated on DNAse agar medium (HiMedia), and the DNase activity was assessed by the presence of a halo after the addition of 1 N HCl to the plate. Gelatin hydrolysis was examined by seeding bacteria on solid medium in test tubes consisting of gelatin (12%), peptone (0.5%), and meat extract (0.3%). For the phenylalanine deaminase test, bacteria were seeded on BHI agar, and after growth, 10% ferric chloride was added to the medium. In the urease test, bacteria were cultured on Christensen’s medium, and for the citrate test, bacteria were cultured on Simmons citrate agar. All tests were performed in duplicate and the evaluations were conducted after 48 h of incubation at 37 °C, except for the gelatin hydrolysis test in which the liquefaction was monitored over a period of two weeks. Additionally, the type strain was subjected to biochemical characterization using the VITEK^®^ 2 system (bioMérieux), according to the manufacturer’s instructions.

Comparative phenotypic analyses were performed using *Moraxella bovis*, *Moraxella bovoculi*, and *Moraxella oculi* strains from our laboratory collection, previously identified by sequencing of the 16–23 S rRNA intergenic spacer (ITS) region. All strains were tested under identical experimental conditions, including the same culture media, incubation temperature, incubation time, and biochemical testing protocols, and assays were performed in parallel to ensure consistency.

### Antimicrobial Susceptibility Testing

Antimicrobial susceptibility testing was performed by the disk diffusion method on Mueller–Hinton agar supplemented with 5% defibrinated sheep blood, following the recommendations of EUCAST (version 15.0, 2025). Bacterial suspensions were adjusted to a 0.5 McFarland standard and inoculated onto agar plates, which were incubated at 35 °C for 18–48 h under aerobic conditions. Inhibition zone diameters were measured in millimeters. As no species-specific clinical breakpoints are currently available for strain 7624LNᵀ or other bovine-associated *Moraxella* species, inhibition zones were interpreted according to the EUCAST clinical breakpoints established for *Moraxella catarrhalis* (v15.0, 2025). When no breakpoint was available, results were reported as not defined (ND). No novel breakpoints were proposed or inferred in this study.

### MALDI-TOF Mass Spectrometry Analysis

Protein extraction was performed according to the protocol described by Freiwald & Sauer [29]. The acquisition and subsequent analysis of mass spectra from the samples were performed according to the methodology described by Bier et al. [30]. MALDI-TOF mass spectra were acquired in positive linear mode, covering a mass/charge ratio (m/z) range between 2,000 and 20,000 Daltons. The acquisition parameters encompassed an IS1 source voltage of 20 kV, IS2 source voltage of 18.55 kV, lens voltage of 8.80 kV, and an ion extraction delay time of 240 ns. Mass spectra from different positions within the well containing the sample were obtained and subsequently summed until reaching a cumulative value of 0.5-1.0 × 10^6^ spectra. Calibration of the system was carried out utilizing the Bacterial Test Standard-BTS, in accordance with the manufacturer’s recommendations (Bruker Daltonics). The mass spectra obtained for the isolates underwent processing using the MALDI Biotyper™ Compass Explorer v.4.1 computer program (Bruker Daltonics) with default settings. For identification based on mass spectra, the m/z signals and their respective intensities obtained for *Moraxella* spp. isolates were compared with the MBT Compass Library BDAL 12,438 from November 2023, containing reference spectra for 15 different *Moraxella* species [31]. Personal isolates of *M. bovis*, *M. bovoculi* genotype 1 and *M. oculi*, previously confirmed through sequencing of the 16–23 S rRNA intergenic region, were included in the database as reference mass spectra for identification of the sampled specimens. Dendrogram analysis was performed using the Biotyper MSP Dendrogram standard method, with distance measure by correlation, single linkage, score oriented and score threshold value of 600 for a single organism.

## Results and Discussion

The draft genome of strain 7624LN^T^ (GCF_035181365.1) comprises 17 scaffolds, with a total length of 1,862,395 bases, DNA G + C content of 41.68%, L50 of 3, and N50 of 314,055. As the genus *Moraxella* exhibits genome sizes ranging from 1,994,386 bp (*M. catarrhalis*) to 3,178,664 bp (*M. lacunata*), and DNA G + C content values range from 39.72% (*M. macacae*) to 47.97% (*M. atlantae*), the genome size and DNA G + C content of strain 7624LNᵀ fall within the overall range observed for the genus, although the reference genome represents the smallest reported for *Moraxella* to date. After quality filtering and trimming, approximately 8.0 million paired-end reads were retained for genome assembly, resulting in an average coverage of approximately 390×. Genome completeness was high (BUSCO bacteria_odb10: C = 99.2% [S = 99.2%, D = 0.0%], F = 0.8%, M = 0.0%; *n* = 124), and no contamination was detected using CheckM (contamination = 0.0%). Genome annotation using RASTtk identified 1,930 CDSs, 4 rRNAs, 43 tRNAs, 29 CRISPR repeats, 28 CRISPR spacers, and one CRISPR array (Table S2). In silico analysis based on PATRIC and Victors databases identified 27 genes associated with antimicrobial resistance and two putative virulence factors (Table S3). These predictions are based on sequence similarity and should be interpreted with caution, as the presence of these genes does not necessarily imply functional activity or phenotypic resistance.

The pairwise GGDC for the strain 7624LN^T^ ranged from 20.5% (*M. oculi*) to 33.7% (*Moraxella atlantae*) when compared to all other known *Moraxella* species, values well below the 70% threshold for species delineation (Table 1). Based on these results, strain 7624LNᵀ is considered to represent a novel species, for which the name *Moraxella tarda* sp. nov. is proposed. Prokaryotic nomenclature was verified using the LPSN database (accessed on 08 May 2026).

**Table 1:** Pairwise comparisons of the genome of strain 7624LN^T^ vs. type strain genomes, calculated by Genome-to-Genome Distance Calculator v. 3.0. The distances were estimated by the formula (sum of all identities found in HSPs divided by overall HSP length)

Species Genome	DDH	Model Confidence Interval	Distance	Prob. DDH ≥ 70%	G + C diff (%)
*Moraxella bovis* genotype 1	22.50	[20.2–24.9%]	0.20	0.00	2.32
*Moraxella bovis* genotype 2	22.80	[20.6–25.3%]	0.19	0.00	2.27
*Moraxella bovoculi* genotype 1	20.60	[18.4–23.0%]	0.21	0.00	3.87
*Moraxella bovoculi* genotype 2	21.70	[19.4–24.1%]	0.20	0.00	3.57
*Moraxella canis*	20.80	[18.6–23.2%]	0.21	0.00	3.38
*Moraxella caprae*	23.40	[21.1–25.9%]	0.19	0.00	2.56
*Moraxella catarrhalis*	22.40	[20.1–24.8%]	0.20	0.00	0.11
*Moraxella caviae*	23.10	[20.8–25.5%]	0.19	0.00	4.84
*Moraxella cuniculi*	22.50	[20.2–24.9%]	0.19	0.00	2.55
*Moraxella equi*	23.50	[21.2–26.0%]	0.19	0.00	2.16
*Moraxella haemolytica*	20.90	[18.7–20.6%]	0.21	0.00	0.91
*Moraxella lacunata*	23.90	[21.6–26.3%]	0.18	0.00	1.90
*Moraxella nasibovis*	21.50	[19.2–23.9%]	0.20	0.00	4.81
*Moraxella nasicaprae*	22.00	[19.7–24.4%]	0.20	0.00	1.96
*Moraxella nasovis*	23.00	[20.7–25.5%]	0.19	0.00	0.44
*Moraxella nonliquefaciens*	22.30	[20.0–24.8%]	0.20	0.00	0.31
*Moraxella oblonga*	21.70	[19.5–24.2%]	0.20	0.00	0.63
*Moraxella oculi*	20.50	[18.3–23.0%]	0.21	0.00	0.83
*Moraxella ovis*	20.80	[18.6–23.3%]	0.21	0.00	3.58
*Moraxella pluranimalium*	21.30	[19.0–23.7%]	0.21	0.00	4.09
*Moraxella porci*	22.10	[19.8–24.5%]	0.20	0.00	4.95

DDH: DNA-DNA Hybridization

**Fig. 1:**
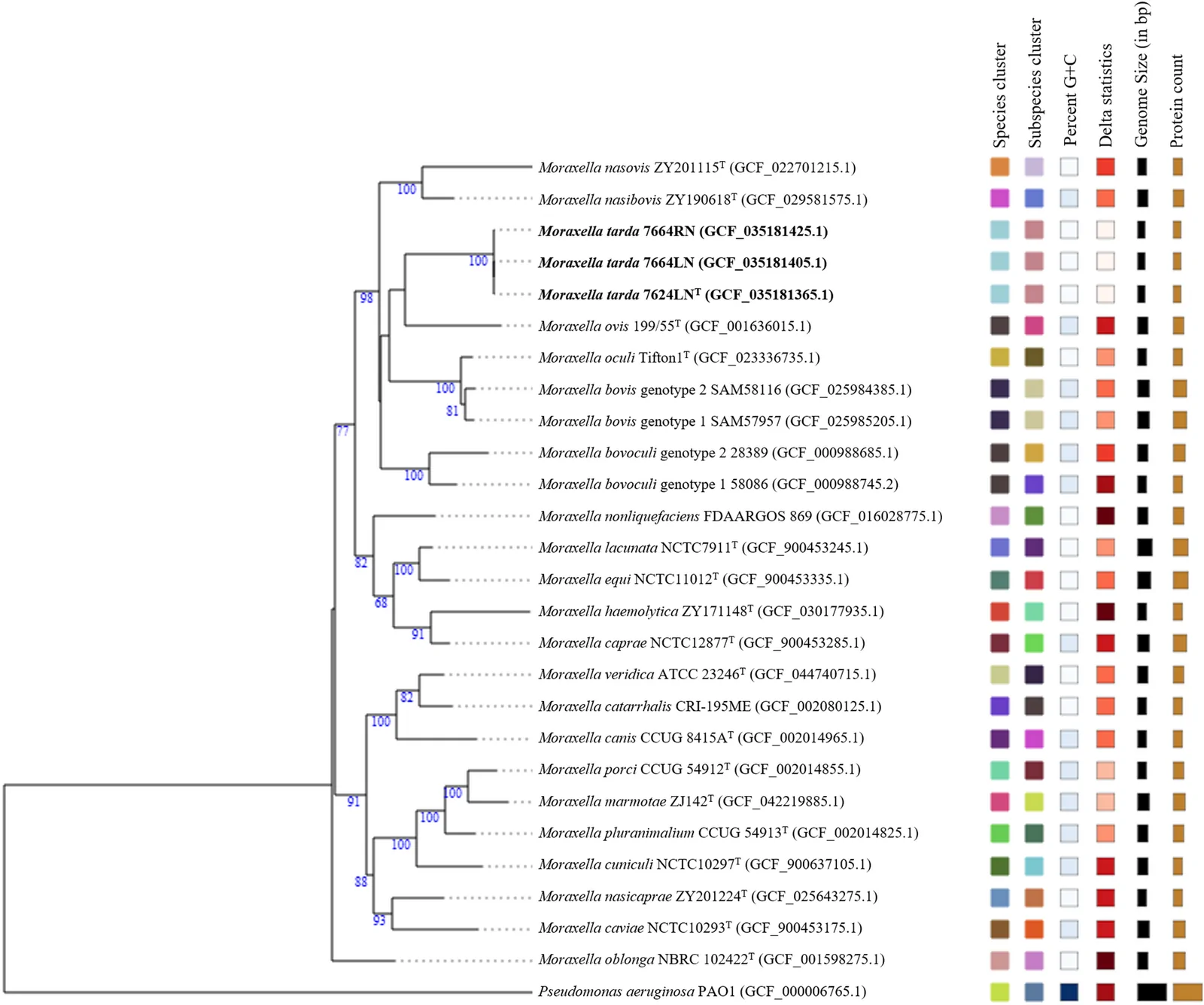
Phylogenetic tree inferred using FastME 2.1.6.1 based on GBDP distances calculated from 16 S rRNA gene sequences. The alignment was generated from full-length 16 S rRNA sequences (~ 1,400 bp). Branch lengths are scaled according to GBDP distances. Numbers above branches indicate pseudo-bootstrap support values (> 80%) based on 100 replicates, with an average branch support of 88.7%. The tree was midpoint-rooted. Leaf labels include affiliation to species and subspecies clusters, genomic G + C content (41.05–66.56%), δ statistics values (0.296–0.417), genome size (1,858,111–6,264,404 bp), number of predicted proteins (1,683–5,681), SSU rRNA gene lengths (1,321–1,615 bp), and strain status (type strain, type species, or user strain)

**Fig. 2:**
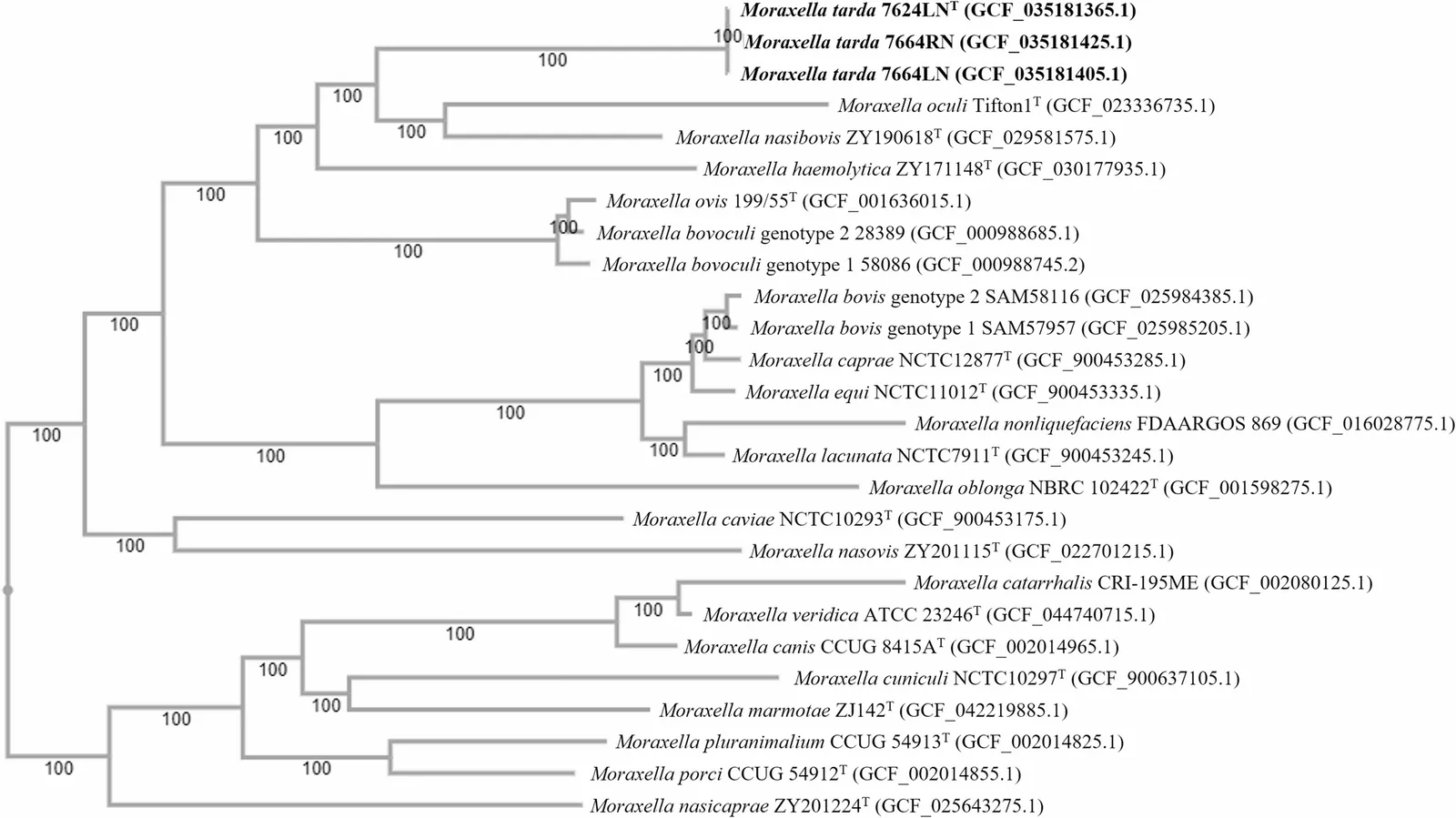
Phylogenetic tree inferred from concatenated alignments of 789 single-copy genes (BV-BRC PGFams) using RAxML. The analysis was performed using a maximum-likelihood approach under the GTR substitution model with gamma correction for rate heterogeneity. Branch support values were estimated using 100 bootstrap replicates and are indicated at each node

The tree inferred with FastME 2.1.6.1 [32] from GDBP distances calculated from 16 S rRNA gene sequences placed strain 7624LN^T^ as a distinct branch, forming a node with *M. nasovis* and *M. nasibovis* (Fig. 1). The ANI values between strain 7624LNᵀ and other *Moraxella* species ranged from 77% to 79% (Table S4). On the other hand, the tree constructed using complete genome data positioned it close to *M. atlantae*, as illustrated in Figure S1. When the comparative analysis was carried out based on the coding genes, strain 7624LNᵀ clustered with the two most recently described bovine-associated species of the genus *Moraxella*, namely *M. nasibovis* and *M. oculi*, forming a monophyletic clade (Fig. 2). This clustering pattern was further supported by an independent phylogenomic reconstruction based on concatenated core genes using EasyCGTree (Figure S2).

Comparative phenotypic characteristics distinguishing strain 7624LNᵀ from closely related bovine-associated *Moraxella* species are summarized in Table S5. The most distinctive features of the strain 7624LNᵀ include its markedly slow growth under standard laboratory conditions and its pinpoint colony morphology after 24 h of incubation, in contrast to the circular colonies observed for *M. bovis*, *M. bovoculi* and *M. oculi*. Additional biochemical characteristics of the type strain determined using the VITEK^®^ 2 system are presented in Table S6.

The antimicrobial susceptibility profile of the type strain is presented in Table S7. According to the EUCAST Clinical Breakpoint Tables v15.0 (2025) for *Moraxella catarrhalis*, inhibition zone diameters obtained for ciprofloxacin (50 mm), erythromycin (48 mm), and tetracycline (37 mm) exceeded the corresponding susceptibility breakpoints (31, 23, and 26 mm, respectively) and were therefore classified as susceptible. For the remaining antimicrobial agents, no applicable EUCAST disk diffusion breakpoints are currently available for *Moraxella catarrhalis*, and inhibition zone diameters are therefore reported without clinical interpretation (ND). No resistance phenotype was detected among the antimicrobial agents for which interpretative criteria were available.

Using the MALDI Biotyper microbial identification system, the three isolates assigned to the novel species matched each other as the top three hits, all with score values greater than 2.7 (Table S8). The next closest identified species was *M. bovoculi*, ranked fourth in the list with a score value of 1.8, well below the 2.3 cutoff value for species-level identification. These results indicate that the protein profiles of the novel isolates are distinct from those of other *Moraxella* species currently represented in the database. Dendrogram analysis based on MALDI-TOF MS protein profiles further supported these findings, as the three isolates assigned to the novel species formed a distinct monophyletic cluster that grouped most closely with *Moraxella bovoculi* isolates (Figure S3).

**Fig. 3:**
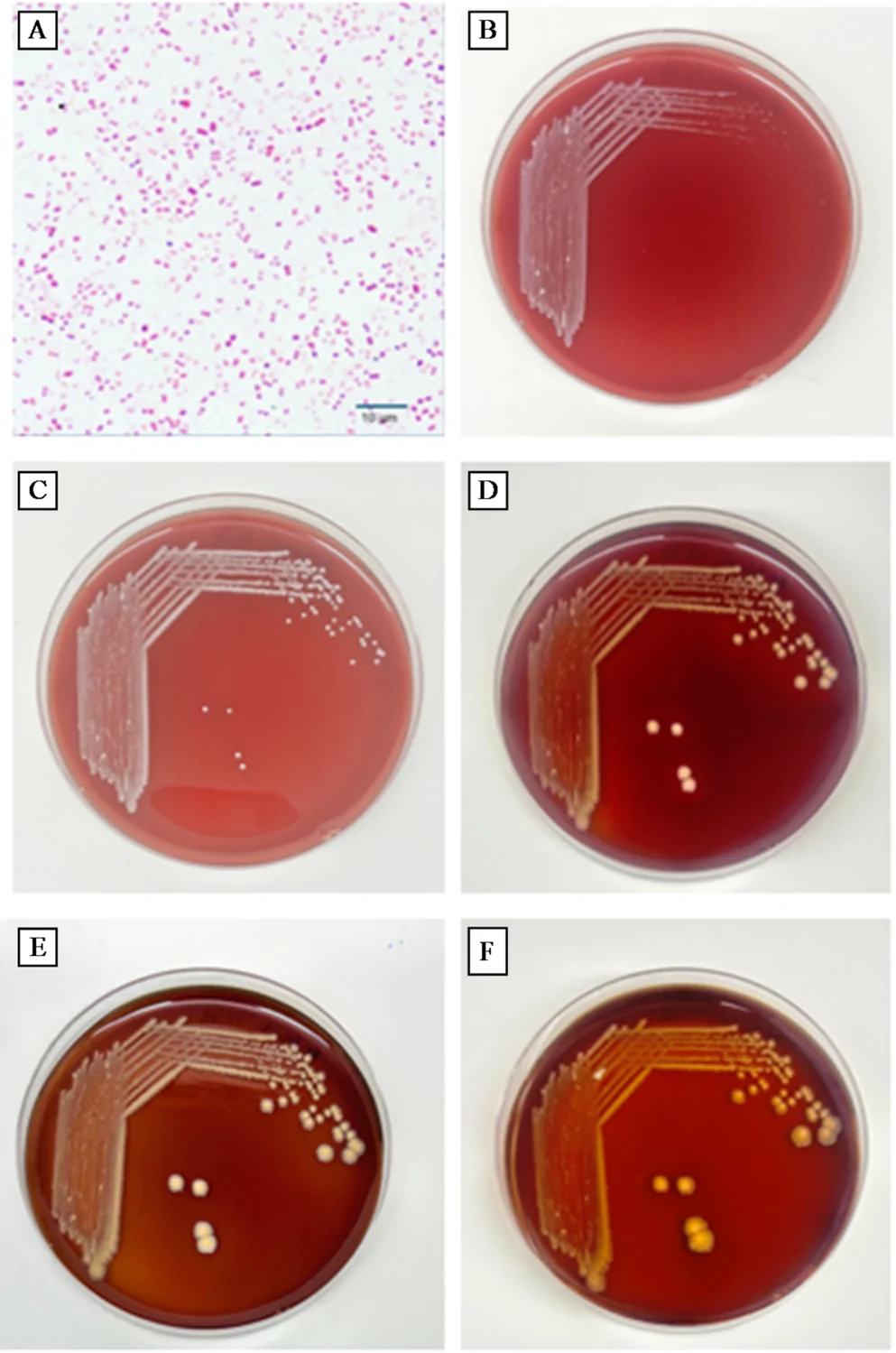
Representative images of cell and colony morphology of strain 7624LN^T^ grown in TSAB medium. **a**: Gram stain. **b-f**: Characteristics of colonies after incubation at 35 °C for 24 h (**b**), 48 h (**c**), 120 h (**d**), 168 h (**e**) and 288 h (**f**)

Cells of strain 7624LNᵀ were Gram-negative, cocci, approximately 0.6–1.0 μm in diameter (Fig. 3a). It grows substantially more slowly than other species of the genus *Moraxella*, such as *M. bovis*, *M. bovoculi* and *M. oculi* in TSAB medium. Colony formation was nearly imperceptible within the initial 24 h of growth, serving as the primary rationale for assigning the specific epithet to this species. After 48 h, colonies became readily visible despite their small size and continue to enlarge for at least seven days after inoculation (Fig. 3b-f). Colonies were small, circular, white to pearly white, convex to papillate, shiny, with entire to wavy margins after 48 h of incubation (Fig. 3c). It lacked hemolytic activity (γ-hemolysis; Fig. 3c-f). Colonies were solid and compact, allowing them to be easily transferred intact across the agar surface using a sterile loop. Growth in BHI broth occurred at temperatures ranging from 25 to 39 °C (optimum 35 °C), at pH values from 6.5 to 8.0 (optimum, 7.5), and in the presence of 0.5-2.0% NaCl (optimum 1.0%). All tests described above were conducted under aerobic conditions, however the strain also exhibited growth under microaerophilic conditions.

Regarding the physiological characterization, strain 7624^T^ exhibited positive reactions for catalase and oxidase but tested negative for motility, hydrogen sulfide production, indole production, phenylalanine deaminase activity, nitrate reduction, glucose, lactose, sucrose, and mannitol fermentation, as well as casein and gelatin hydrolysis. DNase and urease activities were also not detected. Despite the lack of observable motility in SIM medium, genome annotation revealed the presence of pili type IV subunit genes, suggesting the potential for twitching motility. This observation, coupled with specific colony characteristics such as the edge shape and continuous growth, suggested the potential occurrence of twitching motility, akin to other species within the genus *Moraxella*, such as *M. bovis* and *M. catarrhalis* [33, 34]. Further studies are needed to characterize the interaction of strain 7624LN^T^ with its hosts and its potential role in IBK pathogenesis [9, 35].

The recent discovery of new *Moraxella* species in cattle points to the need for the development of more comprehensive molecular identification techniques than currently available methods [36–39] in order to avoid misidentification. Additionally, it is necessary to assess the role of all these species in the ocular and nasopharyngeal microbiota, as well as in the pathogenesis of diseases, particularly IBK.

### Description of *Moraxella tarda* sp. nov

*Moraxella tarda* (tar′da. L. fem. adj. *tarda*, slow, referring to the markedly slow growth of the organism under standard laboratory conditions).

Cells are Gram-stain-negative cocci, 0.6–1.0 μm in diameter, occurring singly, non-motile, oxidase-positive and catalase-positive. Aerobic to microaerophilic and non-hemolytic (γ-hemolysis) on tryptone soy agar supplemented with 5% sheep blood. Colonies are pinpoint to very small after 24 h of incubation at 35 °C, becoming clearly visible after 48 h and continuing to enlarge for at least seven days. Colonies after 48 h are circular, white to pearly white, convex to papillate, shiny, with entire to slightly undulate margins. Colonies are solid and compact, allowing easy transfer across the agar surface using a sterile loop. No growth occurs on MacConkey agar. Growth occurs at temperatures ranging from 25 to 39 °C (optimum 35 °C), at pH 6.5–8.0 (optimum pH 7.5), and in the presence of 0.5–2.0% NaCl (optimum 1.0%). Produces catalase, cytochrome oxidase, glutamyl arylamidase pNA, L-proline arylamidase and tyrosine arylamidase, but does not produce hydrogen sulfide, indole, DNase, urease, or phenylalanine deaminase activity. Does not hydrolyze casein or gelatin and does not ferment glucose, lactose, sucrose or mannitol.

The type strain 7624LNᵀ (= CCP 570ᵀ = IAL 11705ᵀ = LMG 34122ᵀ) was isolated from the nasal cavity of Hereford cattle showing early clinical signs of infectious bovine keratoconjunctivitis on a beef cattle farm located in Dom Pedrito, Rio Grande do Sul, Brazil. The GenBank accession numbers for the 16 S rRNA gene and whole-genome shotgun project are PP069797.1 and JAYGGU000000000, respectively. The genome DNA G + C content of the type strain is 41.7%, with genome size 1.86 Mb.

## Supplementary Information

Below is the link to the electronic supplementary material.

Supplementary Material 1

## Data Availability

Data Availability Statement: The genome sequence of *Moraxella tarda* sp. nov. has been deposited in the NCBI database under accession number GCF_035181365.1. All data generated or analyzed during this study are included in this published article.

## Abbreviations

IBK: Infectious Bovine Keratoconjunctivitis

## Declarations

### Repositories

NCBI Accession numbers: BioSample (SAMN39049954), genome assembly (GCF_035181365.1), 16 S rRNA gene sequence (PP069797.1).
