# Supplementary material for: Moraxella tarda sp. nov., a Novel Species Isolated from Nasal Cavity of Cattle with Infectious Bovine Keratoconjunctivitis
Source: Curr Microbiol. 2026 Jul 9;83(9):467. doi: 10.1007/s00284-026-05050-6 (PMC13350155; doi:10.1007/s00284-026-05050-6)
Supplement: Supplementary file 1 — Supplementary Material 1 [file 284_2026_5050_MOESM1_ESM.docx]

**Supplementary Material**

***Moraxella tarda* sp. nov., isolated from nasal cavity of cattle with Infectious Bovine Keratoconjunctivitis**

Robert Domingues^1, 2^ (ORCID: 0000-0002-4692-8635), Clarissa Vidal de Carvalho^2^ (ORCID: 0009-0004-2336-7427), Daniele Ribeiro de Lima Reis Faza^2, 3^ (ORCID: 0000-0002-5155-7736), Helena Brocardo Comin^4^ (ORCID: 0000-0003-4095-8208), Newton Valério Verbisck^5^ (ORCID: 0000-0001-9817-7223), Alessandra Figueiredo de Castro Nassar^6^ (ORCID: 0000-0001-7490-9186), Fernando Flores Cardoso^1^ (ORCID: 0000-0002-4145-1049), Alessandra Barbosa Ferreira Machado^2^ (ORCID: 0000-0003-3972-7576), Marta Fonseca Martins^3^ (ORCID: 0000-0002-4260-5329), Emanuelle Baldo Gaspar^1^ (ORCID: 0000-0001-7810-5214)

^1^Embrapa Southern Livestock, Bagé, RS, Brazil

^2^Universidade Federal de Juiz de Fora, Juiz de Fora, MG, Brazil

^3^Embrapa Dairy Cattle, Juiz de Fora, MG, Brazil

^4^Universidade Federal do Pampa, Dom Pedrito, RS, Brazil

^5^Embrapa Beef Cattle, Campo Grande, MS, Brazil

^6^Biological Institute, São Paulo Agency for Agribusiness Technology, Secretary of Agriculture and Food Supply, São Paulo, SP, Brazil

Correspondence:

Marta Fonseca Martins, marta.martins@embrapa.br


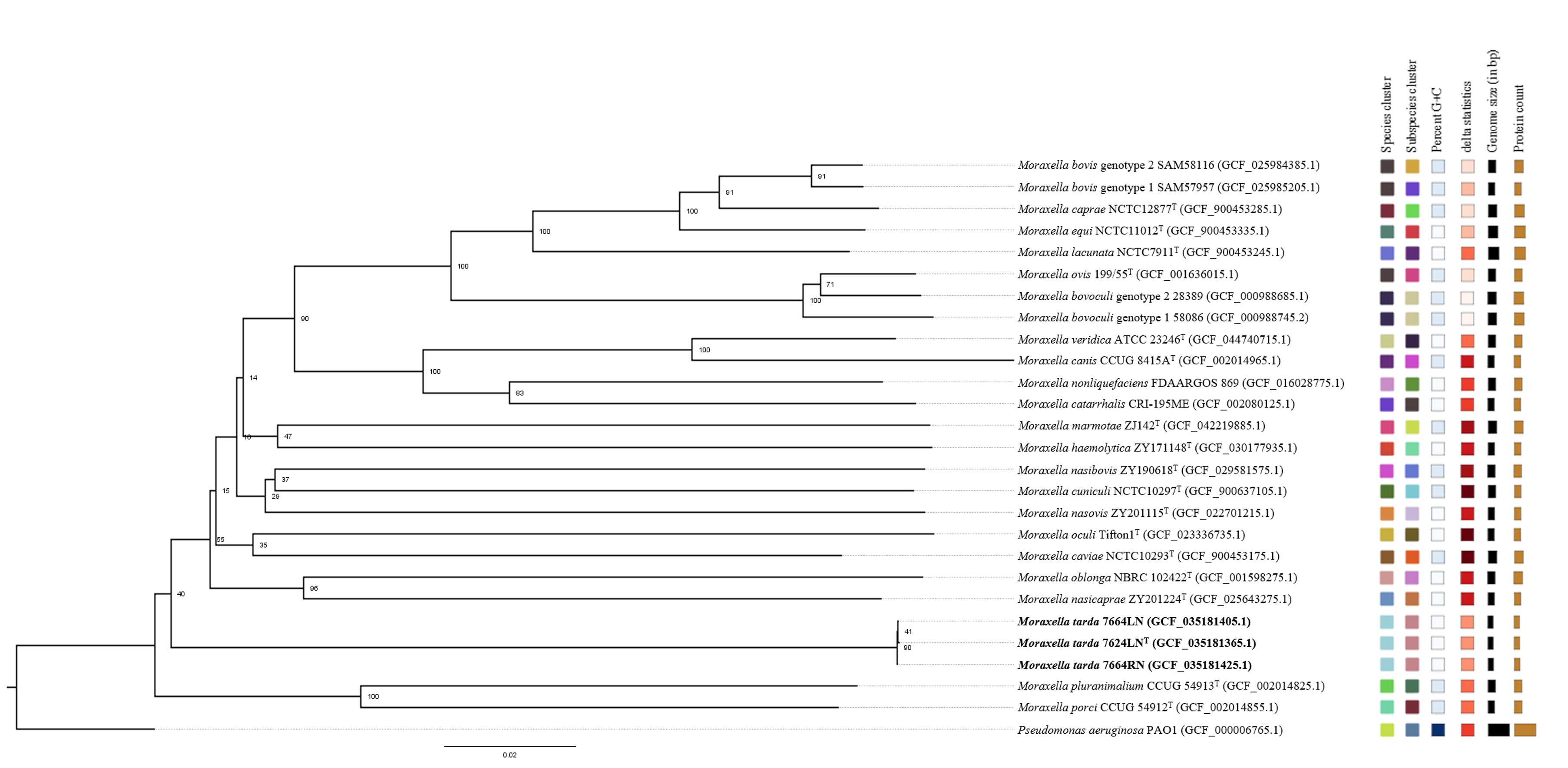


**Figure S1.** Genome-based phylogenetic tree inferred using FastME 2.1.6.1 from GBDP distances (formula d5) calculated from whole-genome sequences. Branch lengths represent GBDP distances. Numbers above branches indicate pseudo-bootstrap support values (>60%) based on 100 replicates, with an average branch support of 52.1%. The tree was midpoint-rooted. Leaf labels include affiliation to species and subspecies clusters, genomic G+C content (41.05–66.56%), δ statistics values (0.212–0.401), genome size (1,858,111–6,264,404 bp), number of predicted proteins (1,683–5,681), SSU rRNA gene lengths (1,321–1,615 bp), and strain status (type strain, type species, or user strain).


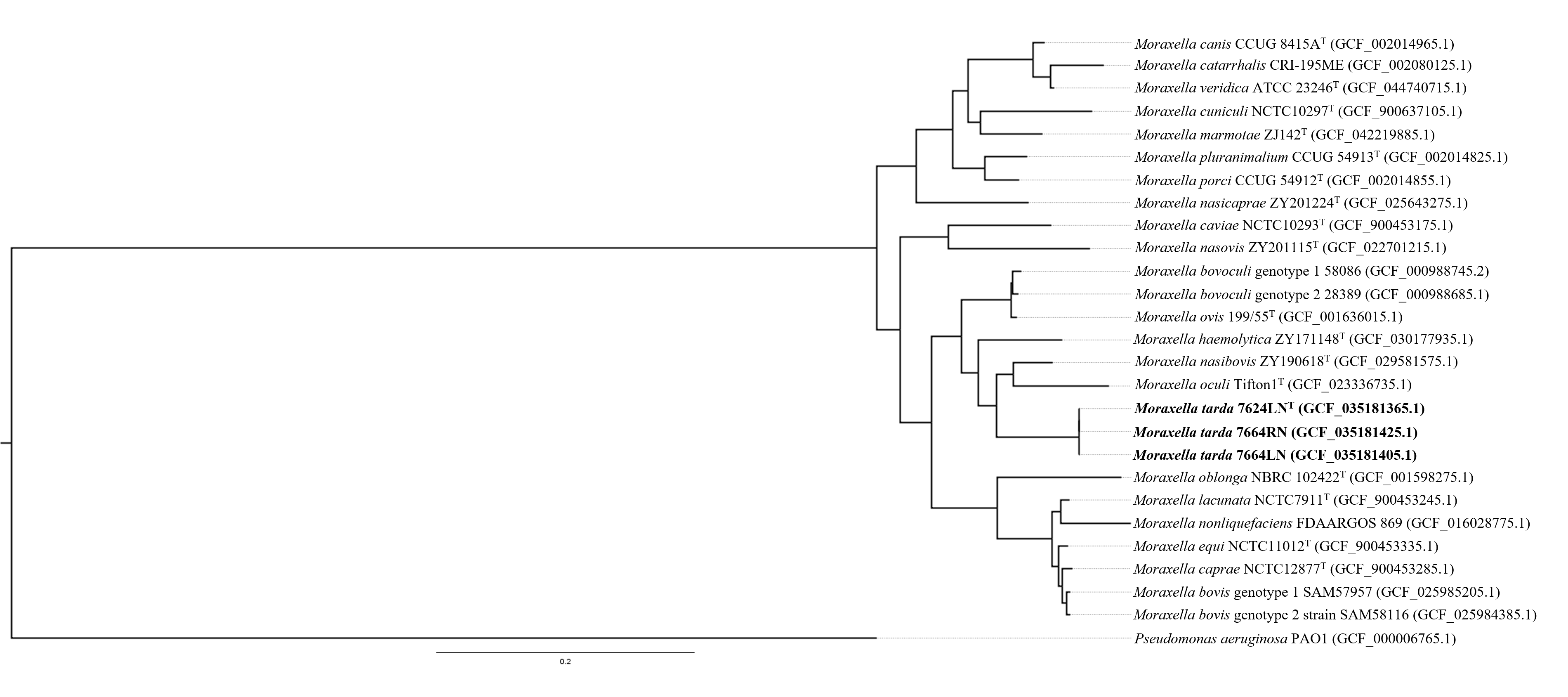


**Figure S2.** Phylogenomic tree of *Moraxella* species inferred from the concatenation of 120 conserved core genes (bac120) using the supermatrix approach implemented in EasyCGTree v4.2. Maximum-likelihood inference was performed with IQ-TREE, and branch support values were estimated using ultrafast bootstrap (1000 replicates). The tree is rooted with *Pseudomonas aeruginosa* as an outgroup. Isolates belonging to *Moraxella tarda* are highlighted in bold. The scale bar indicates the number of substitutions per site.


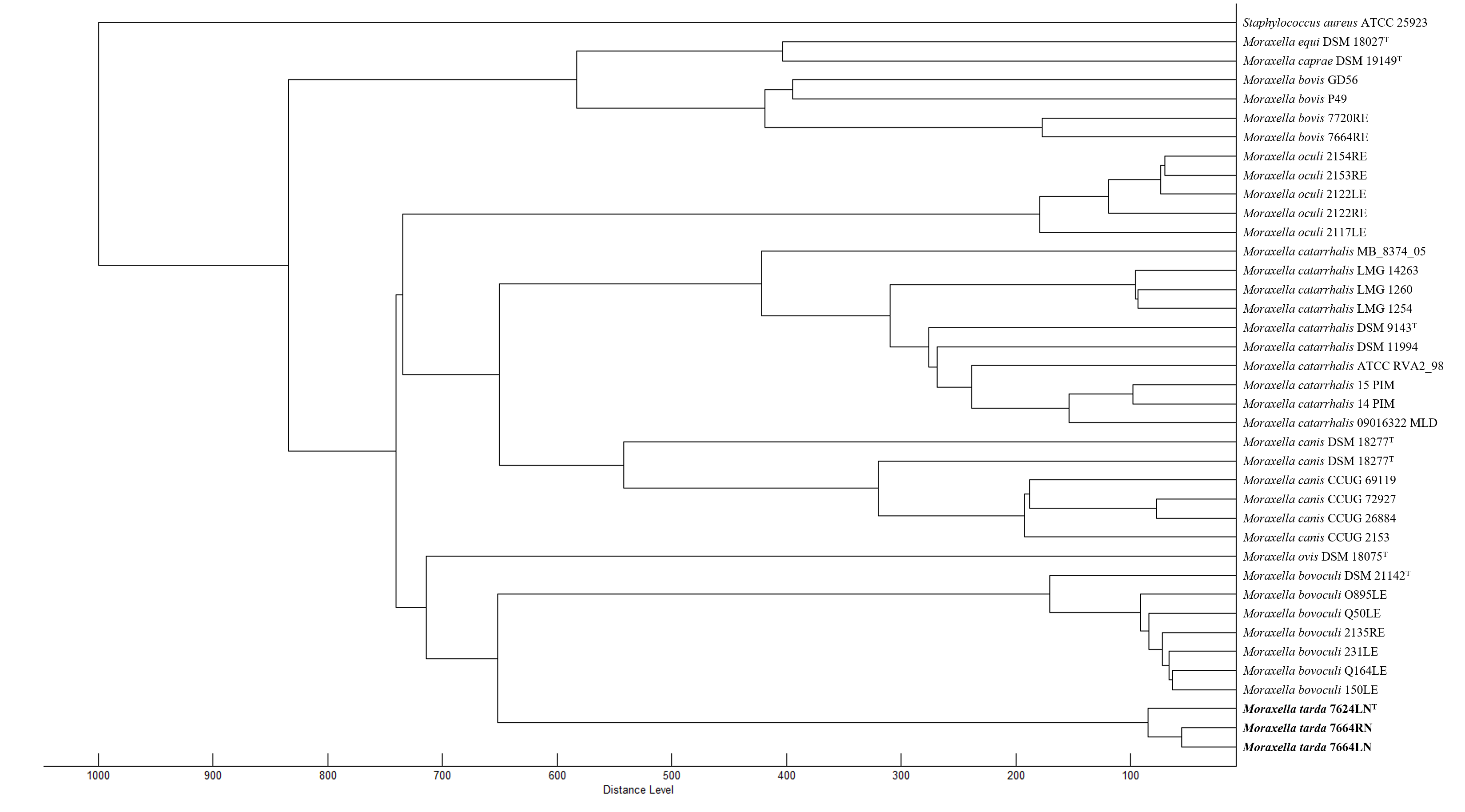


**Figure S3.** Dendrogram generated from MALDI-TOF MS protein profiles using the Biotyper MSP Dendrogram standard method with correlation distance measure, single linkage clustering, score-oriented analysis, and a score threshold value of 600 for a single organism. Type strains are indicated by superscript ^T^.

**Table S1.** Comprehensive listing of species, strains, and their corresponding RefSeq accession numbers utilized in the comparative genomics analyses. The dataset includes all species within the genus *Moraxella* for which genome data is available. In addition, *Pseudomonas* *aeruginosa* was incorporated as outgroup reference.

| **Species** | **Strain** | **RefSeq Accession Number** |
| --- | --- | --- |
| *Moraxella bovis* | SAM57957 (genotype 1) | GCF_025984385.1 |
| *Moraxella bovis* | SAM58116 (genotype 2) | GCF_025985205.1 |
| *Moraxella bovoculi* | 58086 (genotype 1) | GCF_000988745.2 |
| *Moraxella bovoculi* | 28389 (genotype 2) | GCF_000988685.1 |
| *Moraxella canis* | CCUG 8415A^T^ | GCF_002014965.1 |
| *Moraxella caprae* | NCTC12877^T^ | GCF_900453285.1 |
| *Moraxella catarrhalis* | CCRI-195ME | GCF_002080125.1 |
| *Moraxella caviae* | NCTC10293^T^ | GCF_900453175.1 |
| *Moraxella cuniculi* | NCTC10297^T^ | GCF_900637105.1 |
| *Moraxella equi* | NCTC11012^T^ | GCF_900453335.1 |
| *Moraxella haemolytica* | ZY171148^T^ | GCF_030177935.1 |
| *Moraxella lacunata* | NCTC7911^T^ | GCF_900453245.1 |
| *Moraxella marmotae* | ZJ142^T^ | GCF_042219885.1 |
| *Moraxella nasibovis* | ZY190618^T^ | GCF_029581575.1 |
| *Moraxella nasicaprae* | ZY201224^T^ | GCF_025643275.1 |
| *Moraxella nasovis* | ZY201115^T^ | GCF_022701215.1 |
| *Moraxella nonliquefaciens* | FDAARGOS_869 | GCF_016028775.1 |
| *Moraxella oblonga* | NBRC 102422^T^ | GCF_001598275.1 |
| *Moraxella oculi* | Tifton1^T^ | GCF_023336735.1 |
| *Moraxella ovis* | 199/55^T^ | GCF_001636015.1 |
| *Moraxella pluranimalium* | CCUG 54913^T^ | GCF_002014825.1 |
| *Moraxella porci* | CCUG 54912^T^ | GCF_002014855.1 |
| *Moraxella tarda* | 7624LN^T^ | GCF_035181365.1 |
| *Moraxella tarda* | 7664LN | GCF_035181405.1 |
| *Moraxella tarda* | 7664RN | GCF_035181425.1 |
| *Moraxella veridica* | ATCC 23246^T^ | GCF_044740715.1 |
| *Pseudomonas aeruginosa* | PAO1 | GCF_000006765.1 |

**Table S2.** Quantitative and qualitative results of the assembled and annotated genomes for the strain 7624LN^T^ subjected to Illumina sequencing.

| **Attributes** | **Values** |
| --- | --- |
| NCBI Accession number | GCF_035181365.1 |
| Scaffolds | 17 |
| Genome Length (bp) | 1,862,395 |
| DNA G+C content (%) | 41.7 |
| Contig L50 | 3 |
| Contig N50 | 314,055 |
| tRNA | 43 |
| rRNA | 4 |
| CDS | 1,930 |
| BUSCO Completeness (%) | 99.2 |
| CheckM Completeness (%) | 98.2 |
| CheckM Contamination (%) | 0.0 |

**Table S3:** *In silico* predicted antimicrobial resistance and virulence-associated genes identified in the strain 7624LN^T^ based on PATRIC and Victors databases.

| **Detection method** | **Functional category** | **Source** | **Gene** | **Predicted protein product*** | **Classification** | **Antibiotics class** | **PMID** |
| --- | --- | --- | --- | --- | --- | --- | --- |
| BLAT | Virulence Factor | Victors | *hktE* | Catalase KatE |  |  | 7927766 |
| BLAT | Virulence Factor | Victors | *argG* | Argininosuccinate synthase |  |  | 12819083 |
| K-mer Search | Antibiotic Resistance | PATRIC | *katG* | Catalase-peroxidase KatG | antibiotic activation enzyme | Antitubercular drugs | 14638486 |
| K-mer Search | Antibiotic Resistance | PATRIC | *gidB* | 16S rRNA (guanine(527)-N(7))-methyltransferase | gene conferring resistance via absence | Aminoglycosides | 17238915 |
| K-mer Search | Antibiotic Resistance | PATRIC | *tuf* | Translation elongation factor Tu | antibiotic target in susceptible species | Elfamycins | 364475, 9678602 |
| K-mer Search | Antibiotic Resistance | PATRIC | *dxr* | 1-deoxy-D-xylulose 5-phosphate reductoisomerase | antibiotic target in susceptible species | Fosmidomycin | 16321944 |
| K-mer Search | Antibiotic Resistance | PATRIC | *fabG* | 3-hydroxyacyl-CoA dehydrogenase, FabG4 | antibiotic target replacement protein | Triclosan | 24100560, 21081168 |
| K-mer Search | Antibiotic Resistance | PATRIC | *ddl* | D-alanine--D-alanine ligase | antibiotic target in susceptible species | Cycloserine | 24303782, 24033232 |
| K-mer Search | Antibiotic Resistance | PATRIC | *kasA* | 3-oxoacyl-[acyl-carrier-protein] synthase, KASII | antibiotic target in susceptible species | Isoniazid, Triclosan | 10428945 |
| K-mer Search | Antibiotic Resistance | PATRIC | *folA, mfr* | Dihydrofolate reductase | antibiotic target in susceptible species | Diaminopyrimidines | 20169085, 25288078 |
| K-mer Search | Antibiotic Resistance | PATRIC | *gyrB* | DNA gyrase subunit B | antibiotic target in susceptible species | Fluoroquinolones Quinolones Quinolines, Aminocoumarin antibiotics | 21693461, 22279180, 9293187 |
| K-mer Search | Antibiotic Resistance | PATRIC | *macB* | Macrolide export ATP-binding/permease protein MacB | efflux pump conferring antibiotic resistance | Macrolides | 11544226 |
| K-mer Search | Antibiotic Resistance | PATRIC | *murA* | UDP-N-acetylglucosamine 1-carboxyvinyltransferase | antibiotic target in susceptible species | Fosfomycin | 8994972 |
| K-mer Search | Antibiotic Resistance | PATRIC | *rpoB* | DNA-directed RNA polymerase beta subunit | antibiotic target in susceptible species | Rifamycins, Peptide antibiotics | 3050121, 15047531, 16723576 |
| K-mer Search | Antibiotic Resistance | PATRIC | *htdX* | Probable (3R)-hydroxyacyl-CoA dehydratase HtdX | antibiotic target replacement protein |  | 19136596, 24100560, 20511508, 15247240 |
| K-mer Search | Antibiotic Resistance | PATRIC | *rpsL* | SSU ribosomal protein S12p (S23e) | antibiotic target in susceptible species | Aminoglycosides | 7934937 |
| K-mer Search | Antibiotic Resistance | PATRIC | *macA* | Macrolide-specific efflux protein MacA | efflux pump conferring antibiotic resistance | Macrolides | 11544226 |
| K-mer Search | Antibiotic Resistance | PATRIC | *gyrA* | DNA gyrase subunit A | antibiotic target in susceptible species | Fluoroquinolones Quinolones Quinolines | 9293187 |
| K-mer Search | Antibiotic Resistance | PATRIC | *rpoC* | DNA-directed RNA polymerase beta' subunit | antibiotic target in susceptible species | Myxopyronins Corallopyronins, Peptide antibiotics | 16723576 |
| K-mer Search | Antibiotic Resistance | PATRIC | *inhA, fabI* | Enoyl-[acyl-carrier-protein] reductase [NADH] | antibiotic target in susceptible species | Antitubercular drugs, Ethionamide, Triclosan | 18193820, 10869170, 8284673 |
| K-mer Search | Antibiotic Resistance | PATRIC | *oxyR* | Hydrogen peroxide-inducible genes activator | regulator modulating expression of antibiotic resistance genes | Antitubercular drugs | 25799046 |
| K-mer Search | Antibiotic Resistance | PATRIC | *ileS* | Isoleucyl-tRNA synthetase | antibiotic target in susceptible species | Mupirocin | 7929087 |
| K-mer Search | Antibiotic Resistance | PATRIC | *folP* | Dihydropteroate synthase | antibiotic target in susceptible species | Sulfonamides | 15673783 |
| K-mer Search | Antibiotic Resistance | PATRIC | *pgsA* | CDP-diacylglycerol--glycerol-3-phosphate 3-phosphatidyltransferase | protein altering cell wall charge conferring antibiotic resistance | Peptide antibiotics | 22238576 |
| K-mer Search | Antibiotic Resistance | PATRIC | *rpsJ* | SSU ribosomal protein S10p (S20e) | antibiotic target in susceptible species | Tetracyclines, Glycylcyclines | 26124155 |
| K-mer Search | Antibiotic Resistance | PATRIC | *rho* | Transcription termination factor Rho | antibiotic target in susceptible species | Bicyclomycins | 8466900 |
| K-mer Search | Antibiotic Resistance | PATRIC | *fusA* | Translation elongation factor G | antibiotic target in susceptible species | Fusidic acid | 17980694 |
| K-mer Search | Antibiotic Resistance | PATRIC | *alr* | Alanine racemase | antibiotic target in susceptible species | Cycloserine | 19748470, 24303782 |

*Predictions are based on sequence similarity using PATRIC/Victors databases and were not experimentally validated. The presence of these genes does not necessarily imply phenotypic resistance or functional activity. Some genes may be incomplete, non-functional, or associated with antibiotics not evaluated in this study

**Table S4.** Matrix of pairwise average nucleotide identity (ANI) values calculated using FastANI v1.34 with a minimum alignment fraction of 0.1.

|  | *Moraxella bovis* g1 | *Moraxella bovis*  g2 | *Moraxella bovoculi*  g1 | *Moraxella bovoculi*  g2 | *Moraxella canis* | *Moraxella caprae* | *Moraxella catarrhalis* | *Moraxella caviae* | *Moraxella cuniculi* | *Moraxella equi* | *Moraxella haemolytica* | *Moraxella lacunata* | *Moraxella marmotae* | *Moraxella nasibovis* | *Moraxella nasicaprae* | *Moraxella nasovis* | *Moraxella nonliquefaciens* | *Moraxella oblonga* | *Moraxella oculi* | *Moraxella ovis* | *Moraxella pluranimalium* | *Moraxella porci* | *Moraxella* sp. 7624LN ^T^ | *Moraxella veridica* |
| --- | --- | --- | --- | --- | --- | --- | --- | --- | --- | --- | --- | --- | --- | --- | --- | --- | --- | --- | --- | --- | --- | --- | --- | --- |
| *Moraxella bovis* genotype 1 SAM57957 | 100 | 98.37 | 85.12 | 84.20 | 77.51 | 95.58 | 78.89 | 78.22 | 78.35 | 94.61 | 79.28 | 90.33 | 77.95 | 80.02 | 78.76 | 78.71 | 83.91 | 79.45 | 79.23 | 84.32 | 77.68 | 77.56 | 78.43 | 79.05 |
| *Moraxella bovis* genotype 2 SAM58116 | 98.36 | 100 | 85.40 | 84.45 | 77.47 | 95.29 | 78.92 | 77.93 | 78.15 | 94.58 | 79.65 | 90.32 | 77.86 | 80.10 | 78.98 | 78.41 | 83.88 | 79.43 | 79.53 | 84.60 | 77.70 | 77.97 | 78.90 | 78.82 |
| *Moraxella bovoculi* genotype 1 58086 | 85.14 | 85.33 | 100 | 96.48 | 78.00 | 84.65 | 79.22 | 78.70 | 78.44 | 84.09 | 79.25 | 83.54 | 78.51 | 80.18 | 78.88 | 79.04 | 80.13 | 78.24 | 81.72 | 96.13 | 77.92 | 78.05 | 78.71 | 78.90 |
| *Moraxella bovoculi* genotype 2 28389 | 84.41 | 84.72 | 96.34 | 100 | 78.28 | 85.63 | 79.22 | 78.41 | 78.35 | 83.55 | 80.72 | 83.18 | 78.65 | 80.05 | 79.61 | 78.80 | 80.44 | 78.93 | 79.96 | 97.05 | 78.04 | 78.06 | 78.97 | 79.02 |
| *Moraxella canis* CCUG 8415A^T^ | 77.66 | 77.56 | 78.40 | 78.15 | 100 | 77.92 | 83.71 | 78.40 | 78.42 | 77.77 | 77.97 | 78.34 | 79.10 | 78.17 | 78.30 | 77.86 | 78.24 | 77.38 | 78.07 | 78.81 | 78.55 | 79.28 | 77.61 | 93.10 |
| *Moraxella caprae* NCTC12877^T^ | 95.52 | 95.38 | 84.83 | 86.13 | 77.74 | 100 | 78.70 | 78.19 | 78.41 | 93.82 | 79.06 | 90.57 | 77.79 | 80.04 | 79.43 | 78.91 | 84.25 | 79.56 | 78.93 | 85.48 | 77.57 | 77.69 | 78.58 | 78.28 |
| *Moraxella catarrhalis* CRI-195ME | 78.78 | 78.95 | 79.48 | 79.39 | 83.78 | 78.92 | 100 | 78.44 | 78.63 | 79.06 | 78.35 | 81.78 | 79.14 | 78.37 | 78.91 | 78.35 | 84.08 | 77.38 | 77.90 | 79.45 | 78.08 | 78.52 | 77.86 | 90.33 |
| *Moraxella caviae* NCTC10293^T^ | 78.27 | 78.14 | 78.98 | 78.55 | 78.29 | 78.58 | 78.57 | 100 | 78.58 | 78.17 | 78.45 | 78.09 | 78.72 | 79.23 | 78.64 | 78.94 | 77.95 | 77.47 | 78.09 | 78.80 | 78.25 | 78.17 | 77.75 | 78.74 |
| *Moraxella cuniculi* NCTC10297^T^ | 78.10 | 78.01 | 78.60 | 78.19 | 78.33 | 78.24 | 78.46 | 78.11 | 100 | 77.89 | 78.72 | 78.14 | 79.52 | 78.79 | 78.77 | 78.14 | 78.34 | 77.32 | 78.07 | 78.85 | 78.89 | 78.95 | 77.88 | 78.56 |
| *Moraxella equi* NCTC11012^T^ | 94.57 | 94.49 | 83.88 | 83.64 | 77.57 | 93.76 | 78.70 | 78.05 | 78.31 | 100 | 79.24 | 90.57 | 78.05 | 80.61 | 79.11 | 78.57 | 83.78 | 79.49 | 78.42 | 83.89 | 77.40 | 77.39 | 78.29 | 78.68 |
| *Moraxella haemolytica* ZY171148^T^ | 79.36 | 79.38 | 79.24 | 80.64 | 77.80 | 79.10 | 78.18 | 78.34 | 78.40 | 79.28 | 100 | 78.99 | 78.44 | 79.98 | 79.05 | 78.67 | 78.31 | 78.04 | 79.24 | 80.89 | 77.92 | 77.98 | 79.39 | 78.18 |
| *Moraxella lacunata* NCTC7911^T^ | 90.32 | 90.37 | 83.33 | 83.54 | 78.10 | 90.45 | 81.66 | 78.12 | 78.52 | 90.28 | 79.09 | 100 | 77.79 | 79.08 | 78.37 | 78.70 | 87.65 | 78.70 | 78.63 | 83.41 | 77.41 | 77.34 | 78.22 | 80.23 |
| *Moraxella marmotae* ZJ142^T^ | 78.11 | 78.04 | 78.79 | 78.52 | 78.88 | 78.06 | 78.71 | 78.68 | 79.71 | 77.82 | 78.25 | 77.72 | 100 | 78.69 | 79.06 | 78.19 | 77.76 | 77.45 | 77.59 | 78.29 | 79.75 | 79.62 | 77.86 | 79.12 |
| *Moraxella nasibovis* ZY190618^T^ | 80.08 | 79.99 | 80.39 | 80.04 | 78.02 | 80.02 | 78.63 | 79.17 | 78.66 | 80.39 | 79.89 | 79.25 | 78.86 | 100 | 79.28 | 78.69 | 78.00 | 79.05 | 79.37 | 80.41 | 78.79 | 78.68 | 79.24 | 78.67 |
| *Moraxella nasicaprae* ZY201224^T^ | 78.75 | 78.67 | 79.04 | 79.65 | 78.39 | 79.68 | 78.60 | 78.58 | 78.86 | 79.09 | 78.79 | 78.56 | 78.98 | 79.44 | 100 | 78.68 | 78.00 | 80.77 | 78.49 | 78.88 | 78.76 | 78.74 | 78.15 | 79.12 |
| *Moraxella nasovis* ZY201115^T^ | 78.63 | 78.32 | 79.01 | 78.65 | 77.60 | 78.53 | 78.31 | 78.36 | 78.49 | 78.43 | 78.37 | 78.43 | 78.30 | 78.56 | 78.11 | 100 | 78.24 | 77.46 | 77.56 | 78.77 | 78.22 | 77.43 | 78.02 | 78.01 |
| *Moraxella nonliquefaciens* FDAARGOS 869 | 84.16 | 84.07 | 80.15 | 80.47 | 78.17 | 84.25 | 84.43 | 78.12 | 78.34 | 84.02 | 78.47 | 87.92 | 77.80 | 78.31 | 77.99 | 79.01 | 100 | 77.97 | 77.89 | 80.42 | 77.81 | 77.37 | 78.05 | 82.09 |
| *Moraxella oblonga* NBRC 102422^T^ | 79.11 | 79.36 | 78.03 | 78.95 | 77.38 | 79.49 | 77.34 | 77.54 | 77.67 | 79.45 | 78.02 | 78.80 | 77.32 | 78.86 | 80.47 | 77.59 | 77.98 | 100 | 78.08 | 78.67 | 77.15 | 77.33 | 78.12 | 77.38 |
| *Moraxella oculi* Tifton1^T^ | 79.66 | 79.68 | 81.62 | 79.93 | 78.19 | 78.94 | 77.53 | 77.86 | 78.11 | 78.69 | 79.59 | 78.92 | 77.45 | 79.43 | 78.27 | 77.62 | 77.73 | 78.38 | 100 | 80.30 | 77.73 | 77.81 | 78.90 | 78.21 |
| *Moraxella ovis* 199/55^T^ | 84.31 | 84.70 | 95.94 | 97.05 | 78.38 | 85.48 | 79.43 | 78.70 | 78.88 | 83.95 | 80.68 | 83.29 | 78.29 | 80.51 | 79.16 | 78.59 | 80.31 | 78.72 | 80.46 | 100 | 78.45 | 78.34 | 78.70 | 79.07 |
| *Moraxella pluranimalium* CCUG 54913^T^ | 77.48 | 77.60 | 78.13 | 78.36 | 78.58 | 77.53 | 78.16 | 78.32 | 78.77 | 77.44 | 77.69 | 77.25 | 79.74 | 78.90 | 78.62 | 77.93 | 77.68 | 76.92 | 78.00 | 78.24 | 100 | 85.91 | 77.48 | 78.60 |
| *Moraxella porci* CCUG 54912^T^ | 77.66 | 77.76 | 78.15 | 78.44 | 79.07 | 77.66 | 78.41 | 78.16 | 79.11 | 77.48 | 78.08 | 77.46 | 79.66 | 78.87 | 78.54 | 77.34 | 77.52 | 77.28 | 77.81 | 78.37 | 86.11 | 100 | 77.90 | 78.58 |
| *Moraxella* sp. 7624LN ^T^ | 78.25 | 78.89 | 78.80 | 79.07 | 77.52 | 78.82 | 77.96 | 77.85 | 77.88 | 78.54 | 79.24 | 78.50 | 77.77 | 79.48 | 78.39 | 78.28 | 77.85 | 78.10 | 79.17 | 78.99 | 77.62 | 77.68 | 100 | 78.28 |
| *Moraxella veridica* ATCC 23246^T^ | 78.82 | 78.83 | 79.20 | 78.89 | 93.06 | 78.94 | 90.31 | 78.60 | 78.90 | 78.77 | 78.51 | 80.46 | 79.17 | 78.77 | 79.31 | 77.81 | 82.10 | 76.93 | 78.41 | 79.20 | 78.66 | 78.83 | 77.93 | 100 |

**Table S5.** Differential phenotypic characteristics of strain 7624LNᵀ and closely related bovine-associated *Moraxella* species. All strains were isolated from cattle and analyzed in parallel under identical laboratory conditions, including culture media, incubation temperature, incubation time, and biochemical testing protocols.

| **Characteristic** | **strain 7624LNᵀ** | ***Moraxella bovis* strain 6477_OD** | ***Moraxella bovoculi* strain 5623_OE** | ***Moraxella oculi* strain 2117LN** |
| --- | --- | --- | --- | --- |
| Cell morphology | Cocci | Coccobacilli | Cocci | Coccobacilli |
| Gram-stain | Negative | Negative | Negative | Negative |
| Motility | Non-motile | Non-motile | Non-motile | Non-motile |
| Hemolysis on blood agar | γ-hemolysis | β-hemolysis | β-hemolysis | γ-hemolysis |
| Colony morphology (24 h, TSAB) | Pinpoint / very small | Circular | Circular | Circular |
| Growth rate under standard conditions | Slow | Moderate | Moderate | Moderate |
| Growth on MacConkey agar | No growth | No growth | No growth | No growth |
| Oxidase | Produced | Produced | Produced | Produced |
| Catalase | Produced | Produced | Produced | Produced |

**Table S6.** Biochemical characteristics of strain 7624LNᵀ determined using the VITEK® 2 system (bioMérieux). Results are expressed as positive (+) or negative (−) reactions.

| **Test** | **Abbreviation** | **Amount per well** | **Result** |
| --- | --- | --- | --- |
| Alanine–phenylalanine–proline arylamidase activity | APPA | 0.0384 mg | - |
| Adonitol fermentation | ADO | 0.1875 mg | - |
| L-Pyrrolidonyl arylamidase activity | PyrA | 0.0180 mg | - |
| L-Arabitol fermentation | lARL | 0.3000 mg | - |
| D-Cellobiose fermentation | dCEL | 0.3000 mg | - |
| β-Galactosidase activity | BGAL | 0.0360 mg | - |
| Hydrogen sulfide production | H2S | 0.0024 mg | - |
| β-N-Acetylglucosaminidase activity | BNAG | 0.0408 mg | - |
| Glutamyl arylamidase pNA activity | AGLTp | 0.0324 mg | + |
| D-Glucose fermentation | dGLU | 0.3000 mg | - |
| Gamma-glutamyl transferase activity | GGT | 0.0228 mg | - |
| Glucose fermentation | OFF | 0.4500 mg | - |
| β-Glucosidase activity | BGLU | 0.0360 mg | - |
| D-Maltose fermentation | dMAL | 0.3000 mg | - |
| D-Mannitol fermentation | dMAN | 0.1875 mg | - |
| D-Mannose fermentation | dMNE | 0.3000 mg | - |
| β-Xylosidase activity | BXYL | 0.0324 mg | - |
| β-Alanine arylamidase pNA activity | BAlap | 0.0174 mg | - |
| L-Proline arylamidase activity | ProA | 0.0234 mg | + |
| Lipase activity | LIP | 0.0192 mg | - |
| Palatinose fermentation | PLE | 0.3000 mg | - |
| Tyrosine arylamidase activity | TyrA | 0.0276 mg | + |
| Urease activity | URE | 0.1500 mg | - |
| D-Sorbitol fermentation | dSOR | 0.1875 mg | - |
| Sucrose fermentation | SAC | 0.3000 mg | - |
| D-Tagatose fermentation | dTAG | 0.3000 mg | - |
| D-Trehalose fermentation | dTRE | 0.3000 mg | - |
| Citrate (sodium) utilization | CIT | 0.0540 mg | - |
| Malonate utilization | MNT | 0.1500 mg | - |
| 5-Keto-D-gluconate fermentation | 5KG | 0.3000 mg | - |
| Alkalinization of L-lactate | lLATk | 0.1500 mg | - |
| α-Glucosidase activity | AGLU | 0.0360 mg | - |
| Alkalinization of succinate | SUCT | 0.1500 mg | - |
| β-N-Acetylgalactosaminidase activity | NAGA | 0.0306 mg | - |
| α-Galactosidase activity | AGAL | 0.0360 mg | - |
| Phosphatase activity | PHOS | 0.0504 mg | - |
| Glycine arylamidase activity | GlyA | 0.0120 mg | - |
| Ornithine decarboxylase activity | ODC | 0.3000 mg | - |
| Lysine decarboxylase activity | LDC | 0.1500 mg | - |
| Assimilation of L-histidine | IHISa | 0.0870 mg | - |
| Fumarate utilization | FUM | 0.1260 mg | - |
| β-Glucuronidase activity | BGUR | 0.0378 mg | - |
| Resistance to O/129 (vibriostat) | O129R | 0.0105 mg | - |
| Glu–Gly–Arg arylamidase activity | GGAA | 0.0576 mg | - |
| Assimilation of L-malate | IMLTa | 0.0420 mg | - |
| Ellman reaction | ELLM | 0.0300 mg | - |
| Assimilation of L-lactate | ILATa | 0.1860 mg | - |

**Table S7.** Antimicrobial susceptibility profile of strain 7624LNᵀ determined by disk diffusion assay. Inhibition zone diameters (mm) are presented for each antimicrobial agent tested.

| **Antimicrobial agent** | **Disk content** | **Inhibition zone diameter (mm)** | **EUCAST Interpretation*** | **Breakpoint (mm)**** |
| --- | --- | --- | --- | --- |
| Amikacin | 30 µg | 39 | - | ND |
| Amoxicillin | 10 µg | 53 | - | ND |
| Ampicillin | 10 µg | 52 | - | ND |
| Ampicillin–sulbactam | 20 µg | 54 | - | ND |
| Cephalexin | 30 µg | 48 | - | ND |
| Cephalothin | 30 µg | 56 | - | ND |
| Chloramphenicol | 30 µg | 43 | - | ND |
| Ciprofloxacin | 5 µg | 50 | S | 31 |
| Doxycycline | 30 µg | 38 | - | ND |
| Enrofloxacin | 5 µg | 46 | - | ND |
| Erythromycin | 15 µg | 48 | S | 23 |
| Gentamicin | 10 µg | 42 | - | ND |
| Kanamycin | 30 µg | 43 | - | ND |
| Nalidixic acid | 30 µg | 34 | - | ND |
| Neomycin | 30 µg | 35 | - | ND |
| Norfloxacin | 10 µg | 50 | - | ND |
| Oxytetracycline | 1 µg | 45 | - | ND |
| Penicillin G | 10 UI | 63 | - | ND |
| Polymyxin B | 300 U | 25 | - | ND |
| Rifampicin | 5 µg | 54 | - | ND |
| Streptomycin | 10 µg | 28 | - | ND |
| Sulfadiazine–trimethoprim | 25 µg | 33 | - | ND |
| Sulfamethoxazole–trimethoprim | 25 µg | 34 | - | ND |
| Sulfonamide | 300 µg | 47 | - | ND |
| Tetracycline | 30 µg | 37 | S | 26 |
| Tobramycin | 10 µg | 38 | - | ND |
| Trimethoprim | 5 µg | 34 | - | ND |
| Vancomycin | 30 µg | 27 | - | ND |

* EUCAST interpretation based on Clinical Breakpoint Tables v15.0 (2025) for *Moraxella catarrhalis*. **S**, susceptible (indicates that the inhibition zone diameter exceeded the corresponding EUCAST susceptibility breakpoint shown in the final column).

** **ND**, not defined (no EUCAST disk diffusion breakpoint available for *Moraxella catarrhalis* in EUCAST v15.0, 2025).
Antimicrobial susceptibility testing was interpreted using EUCAST clinical breakpoints for *Moraxella catarrhalis* (v15.0, 2025), as no species-specific breakpoints are currently available for bovine-associated *Moraxella* species.

**Table S8:** Rate classification results for strain 7624LN^T^ as determined by Bruker Daltonics MALDI Biotyper system. Samples without NCBI number identifier were characterized in this study and had their reference spectra included in the database, after 16S rRNA sequencing confirmation at species level.

| **Rank (Quality)** | **Matched Pattern** | **Score Value** | **NCBI Identifier** |
| --- | --- | --- | --- |
| 1 | *Moraxella* sp. 7624LN^T^ | 2.836 | - |
| 2 | *Moraxella* sp. 7664LN | 2.803 | - |
| 3 | *Moraxella* sp. 7664RN | 2.789 | - |
| 4 | *Moraxella bovoculi* 2135RE | 1.801 | - |
| 5 | *Acinetobacter radioresistens* UFL B381 | 1.793 | 40216 |
| 6 | *Moraxella bovoculi* DSM 21142^T^ | 1.776 | 386891 |
| 7 | *Aromatoleum pretroleum* MPB ToN1 | 1.772 | 12960 |
| 8 | *Weissella viridescens* DSM 20410^T^ | 1.768 | 1629 |
| 9 | *Moraxella bovoculi* 150LE | 1.742 | - |
| 10 | *Aromatoleum tolulyticus* MPB Tol4 | 1.733 | 34027 |
| 11 | *Kandleria vitulina* DSM 20405^T^ | 1.724 | 1630 |
| 12 | *Moraxella nonliquefaciens* CIP 100617 | 1.724 | 478 |
| 13 | *Lactobacillus sakei* *carnosus* DSM 15740 | 1.717 | 214325 |
| 14 | *Acinetobacter bereziniae* CIP 70_12T | 1.717 | 469 |
| 15 | *Acinetobacter johnsonii* LMG 1005 | 1.716 | 40214 |
| 16 | *Pseudomonas flavescens* DSM 12071^T^ | 1.713 | 29435 |
| 17 | *Acinetobacter lwoffii* LMG 10596 | 1.705 | 28090 |
| 18 | *Pseudomonas thermotolerans* DSM 14292^T^ | 1.698 | 157784 |
| 19 | *Moraxella oculi* 2122RE | 1.689 | - |
| 20 | *Sinomonas atrocyanea* DSM 20127^T^ | 1.671 | 37927 |
| 21 | *Moraxella oculi* 2153RE | 1.67 | - |
| 22 | *Wolinella succinogenes* DSM 1740^T^ | 1.665 | 844 |
| 23 | *Aromatoleum toluvorans* MPB Td21 | 1.663 | 92002 |
| 24 | *Halomonas elongata* UFL B496 | 1.66 | 2746 |
| 25 | *Lactobacillus sakei* DSM 6333 | 1.655 | 1599 |
| 26 | *Arthrobacter histidinolovorans* DSM 20115^T^ | 1.646 | 43664 |
| 27 | *Lactobacillus delbrueckii* ssp *lactis* DSM 20076 | 1.643 | 29397 |
| 28 | *Lactobacillus sakei* ssp *carnosus* DSM 15831^T^ | 1.642 | 214325 |
| 29 | *Staphylococcus simulans* DSM 20324 | 1.642 | 1286 |
| 30 | *Moraxella nonliquefaciens* PIM 16 | 1.641 | 478 |
